# Prader-Willi syndrome: reflections on seminal studies and future therapies

**DOI:** 10.1098/rsob.200195

**Published:** 2020-09-23

**Authors:** Michael S. Chung, Maéva Langouët, Stormy J. Chamberlain, Gordon G. Carmichael

**Affiliations:** Department of Genetics and Genome Sciences, UCONN Health, 400 Farmington Avenue, Farmington, CT 06030, USA

**Keywords:** Prader-Willi syndrome, imprinting, non-coding RNA, neurodevelopmental disorder, snoRNA, epigenetics

## Abstract

Prader-Willi syndrome (PWS) is caused by the loss of function of the paternally inherited 15q11-q13 locus. This region is governed by genomic imprinting, a phenomenon in which genes are expressed exclusively from one parental allele. The genomic imprinting of the 15q11-q13 locus is established in the germline and is largely controlled by a bipartite imprinting centre. One part, termed the Prader-Willi syndrome imprinting center (PWS-IC), comprises a CpG island that is unmethylated on the paternal allele and methylated on the maternal allele. The second part, termed the Angelman syndrome imprinting centre, is required to silence the PWS_IC in the maternal germline. The loss of the paternal contribution of the imprinted 15q11-q13 locus most frequently occurs owing to a large deletion of the entire imprinted region but can also occur through maternal uniparental disomy or an imprinting defect. While PWS is considered a contiguous gene syndrome based on large-deletion and uniparental disomy patients, the lack of expression of only non-coding RNA transcripts from the *SNURF-SNRPN/SNHG14* may be the primary cause of PWS. Patients with small atypical deletions of the paternal *SNORD116* cluster alone appear to have most of the PWS related clinical phenotypes. The loss of the maternal contribution of the 15q11-q13 locus causes a separate and distinct condition called Angelman syndrome. Importantly, while much has been learned about the regulation and expression of genes and transcripts deriving from the 15q11-q13 locus, there remains much to be learned about how these genes and transcripts contribute at the molecular level to the clinical traits and developmental aspects of PWS that have been observed.

## 1. Overview

Prader-Willi Syndrome (PWS) is a neurodevelopmental disorder with hallmark traits of hypotonia, hypogonadism and hyperphagia/obesity. It affects approximately 1 out of 15 000 live births and currently has no known cure [1–3]. Although patients universally possess the hallmark traits, they can also demonstrate growth hormone deficiency, characteristic facial features, developmental delay and behavioural problems. The onset of various neuroendocrine phenotypes suggests that PWS primarily impacts the hypothalamus although other organs might still be affected. Patients are diagnosed at birth and are followed by endocrinologists throughout their lives. Despite several advancements in clinical care, the majority of individuals with PWS have a life expectancy of 29.5 years with the most common causes of mortality being from respiratory, cardiac and gastrointestinal failures [4].

**Figure 1.**
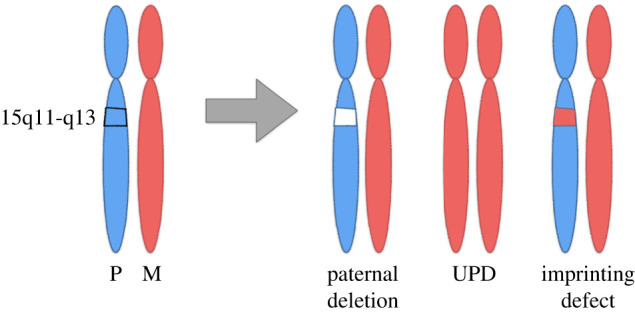
The chr15q11-q13 region is genomically imprinted, with some genes only expressed from the paternal chromosome and some only from the maternal chromosome. Prader-Willi syndrome is associated with loss of expression from the paternal chromosome. This can occur either through paternal deletion, uniparental disomy (UPD) or an imprinting defect (ID).

**Figure 2.**
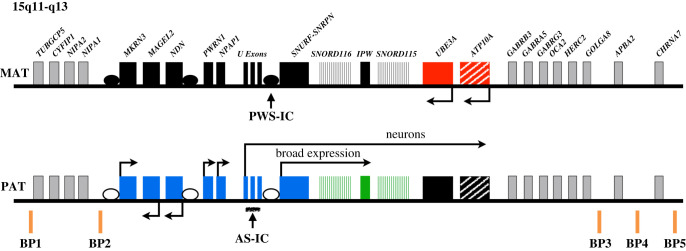
Overview of the chr15q11-q13 region. Genes in blue are expressed only from the paternal chromosome (PAT) and genes in red are expressed only from the maternal chromosome (MAT). Genes in grey are expressed biallelically and those in black are silent imprinted genes. *ATP10A* is partially imprinted (hatched boxes). Expressed non-coding regions are denoted in green. The imprinting centre (PWS-IC) that controls paternal chromosome expression is noted. The Angelman syndrome imprinting centre (AS-IC) lies within the U-exons on the paternal chromosome. Unfilled circles represent unmethylated DMRs and filled circles represent methylated DMRs. PWS patients often contain genetic deletions between breakpoints 1 or 2 (BP1, BP2) and breakpoints 3, 4 or 5 (BP3–5). Transcription is noted by horizontal arrows and is discussed in detail in the text. Not drawn to scale.

PWS is caused by the loss of function of the paternally inherited 15q11-q13 locus. This region is governed by genomic imprinting, a phenomenon in which genes are expressed exclusively from one parental allele. The genomic imprinting of the 15q11-q13 locus is established in the germline and is largely controlled by a bipartite imprinting centre. One part, termed the Prader-Willi syndrome imprinting centre (PWS-IC) [5–7], comprises a CpG island that is unmethylated on the paternal allele and methylated on the maternal allele. The second part, termed the Angelman syndrome imprinting centre (AS-IC), is required to silence the PWS_IC in the maternal germline [8,9]. The loss of the paternal contribution of the imprinted 15q11-q13 locus most frequently occurs owing to a large deletion (LD) of the entire imprinted region (LD approx. 5 Mb, 65%-75%) [10,11] but can also occur through maternal uniparental disomy (UPD, 20%–30%) [12–14] or an imprinting defect (ID) (1%–3%) [15,16] (figure 1). Large deletions typically occur between five common breakpoint regions illustrated in figure 2. While PWS is considered a contiguous gene syndrome based on LD and UPD patients, the lack of expression of only non-coding RNA transcripts from the *SNURF-SNRPN/SNHG14* may be the primary cause of PWS. Patients with small atypical deletions (SD) of the paternal *SNORD116* cluster alone appear to have most of the PWS related clinical phenotypes [17–22]. However, the milder phenotypes present in these patients probably indicate that concurrent absence of other regions in the locus may contribute to greater severity of PWS phenotype. The loss of the maternal contribution of the 15q11-q13 locus causes a separate and distinct condition called Angelman syndrome [23]. Importantly, while much has been learned about the regulation and expression of genes and transcripts deriving from the 15q11-q13 locus, there remains much to be learned about how these genes and transcripts contribute at the molecular level to the clinical traits and developmental aspects of PWS that have been observed.

## 2. Clinical features

### 2.1. Diagnosis

**Figure 3.**
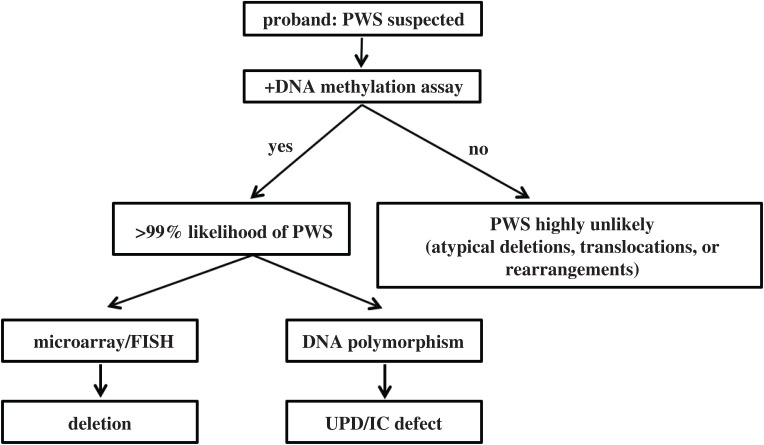
Workflow for PWS diagnosis. See text for details.

Patients suspected of having PWS are often first screened using a DNA methylation assay for the PWS-IC [24–26] (figure 3). Unaffected individuals will show one unmethylated allele and one methylated allele. However, the vast majority of PWS patients will show only a methylated allele. The PWS subtype can be differentiated further through additional assays. The deletion subtype can be identified by chromosomal microarray or DNA fluorescence *in situ* hybridization (FISH). The former assay can also identify the precise deletion breakpoints as well as microdeletions involving the IC and *SNORD116* cluster, depending on the size limitations of the microarray analysis. The UPD and IC defect subtypes are determined by interrogating the parental inheritance of the two chromosome 15 alleles [27]. The parents of the proband are typed to identify their specific microsatellite marker alleles. If the proband has microsatellite marker contribution from one parent, the proband has UPD, whereas contributions from both parents indicate ID. Rare cases of chromosomal rearrangements such as translocations and inversions are detected by using both FISH and chromosomal karyotyping. A very rare cohort of patients with PWS owing to deletions involving the *SNORD116* cluster may not test positive using the DNA methylation test. Therefore, if PWS is suspected and the DNA methylation test is negative, a chromosomal microarray may still be warranted.

Advanced diagnostics tools such as methylation specific-multiplex ligation-dependent probe amplification allow for simultaneous evaluation of DNA methylation and the presence of deletions [28]. The probe targets five different differentially methylated regions (DMR) in the locus and can help identify both IC and *SNORD116* cluster microdeletions. If no deletions are detected, DNA polymorphism assay still must be conducted to differentiate between UPD and ID subtypes.

### 2.2. Nutritional phases

**Figure 4.**
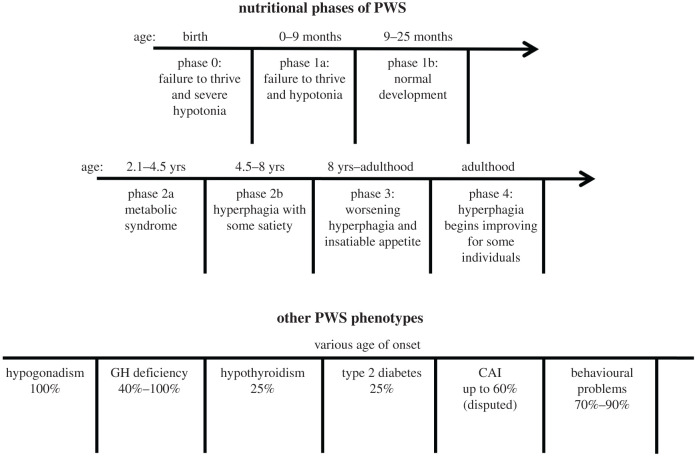
PWS clinical phenotypes, their prevalence and age of onset.

The different stages of PWS can be divided into phases 0–4 based on the onset of specific nutritional phenotypes [29] (figure 4). Seven distinct phases were identified by Miller *et al*. Individuals with PWS demonstrate decreased fetal movements and present at birth with unexplained failure to thrive and severe hypotonia (phase 0). These traits continue in the newborn phase (phase 1a: 0–9 months) and are followed by approximately a 17-month period of normal development (phase 1b: 9–25 months). In the childhood years, PWS patients begin to develop metabolic syndrome with weight gain despite the absence of additional food consumption (phase 2a: 2.1–4.5 years). The patients then begin to develop hyperphagia with some satiety (phase 2b: 4.5–8 years). Through adolescence and into adulthood, the patients' metabolic syndrome and hyperphagia continue to worsen and peaks around this time (phase 3: 8 years–adulthood). The appetite is reported to be impossible to satiate for some patients during this period. The hyperphagic drive begins to decrease and becomes satiable again for some patients (phase 4: adulthood). Many behavioural and cognitive problems that are present through the clinical phases are heavily correlated to the degree of patients’ hyperphagia [30].

### 2.3. Hallmark traits

Hypotonia is most prominent in the neonatal phase. Patients demonstrate severe weakness, poor reflexes, decreased arousal and poor suck/appetite [29,31]. These traits lead to failure to thrive and often require patients to be placed on feeding tubes for various amounts of time. The cause of hypotonia is central in nature (deficiencies in growth hormone (GH), thyroid stimulating hormone, and cortisol) [32] as neuromuscular studies yield insignificant findings [33]. Hypotonia begins to improve once the patients are able to feed themselves and becomes mild in adulthood [29,31]. The lower muscle tone in patients leads to decreased energy expenditure and lower overall caloric requirements.

Hypogonadism is noted at birth and is present throughout the patient's lifetime. Patients of both sexes demonstrate genital hypoplasia, incomplete puberty, underdevelopment of secondary sexual characteristics and infertility later in life [34,35]. Hypogonadism was initially believed to be entirely owing to hypothalamic deficits. However, decrease in levels of hormones directly synthesized by the gonads in non-hypogonadotropic patients indicate that both the hypothalamus and the primary gonads may be involved [35–38].

Obesity accompanied by hyperphagia is perhaps the most recognizable feature of PWS. The degree and onset of hyperphagia and obesity depend on the nutritional phases mentioned in the previous section. PWS patients' food-seeking behaviour is thought to be lack of satiety owing to hypothalamic dysfunction. Both children and adults with PWS have been found to have significantly elevated levels of the orexigneic hormone ghrelin [39–41]. However, short and long-term pharmacological interventions that lowered ghrelin to physiological levels failed to improve hyperphagia and obesity in children (age 11–14) and adults (age 25) [42–44]. Thus it is unlikely that a single pathway controls appetite in PWS patients. The lack of satiety, metabolic syndrome in phase 2a and lower caloric requirements (see hypotonia) are believed to be primary contributors to the observed obesity in patients.

### 2.4. Other endocrinologic traits

PWS is also associated with a number of other traits, including hypothalamic dysfunction, respiratory distress, sleep disturbance, type 2 diabetes, musculoskeletal issues and behavioural problems (figure 4). PWS patients suffer from growth hormone deficiency and demonstrate reduced growth hormone secretion both in childhood and adulthood [32,45]. Patients have short statures in childhood and the absence of a growth spurt during puberty results in more pronounced phenotype in adulthood [46]. Central hypothyroidism is reported in a quarter of children with PWS. The lower circulating levels of T3 and T4 [47,48] are believed to compound patients' symptoms such as hypotonia and obesity. Later in life, the incidence of hypothyroidism drops to comparable levels to that of unaffected populations [49]. Central adrenal insufficiency (CAI) is also noted in some PWS patients. The lower levels of cortisol are believed to disturb the metabolism of carbohydrates, proteins and fats in PWS patients. The precise prevalence of CAI is to be determined as one study reported prevalence up to 60% [50] while others reported much lower prevalence [45,51]. type 2 diabetes is observed in a quarter of PWS patients and is a secondary complication of obesity [52]. However, it is rarely observed in the absence of obesity in PWS patients [47]. Maintenance of an appropriate caloric diet, growth hormone therapy (GHT), and counselling can dramatically reduce the incidence of obesity and type 2 diabetes in PWS patients.

#### 2.4.1. Respiratory distress

Respiratory distress in PWS patients is multifactorial in origin. During a normal physiological response, the hypothalamus helps adjust the respiratory rate to compensate for increase in carbon dioxide and decrease in oxygen levels. However, PWS patients often show an imbalance in this response owing to hypothalamic dysfunction and do not compensate adequately to hypercapnic states [53,54]. The hypotonia associated with PWS can also lead to poor respiratory muscle tone and depressed respiratory response [55]. This feature can lead to increased aspiration and respiratory infection owing to weaker respiratory musculature. Lastly, obesity can lead to obstructive sleep apnea [56].

#### 2.4.2. Sleep disturbances

Alterations in sleep patterns are well reported and can be caused by both hypothalamic dysfunction and respiratory distress [56–59]. Disturbances in crucial hormones such as orexin and acetyl cholinergic neurons in the pedunculo-pontine tegmental nucleus lead to abnormalities in the circadian rhythm, sleep/wake cycles and sleep architecture [57,60,61]. Abnormalities in respiratory response and illnesses such as obstructive sleep apnea can further compound sleep disturbances in patients [57,58].

#### 2.4.3. Behavioural problems

PWS patients demonstrate both hyperphagia related and non-hyperphagia related behavioural problems. Non-hyperphagia related problems include tantrums, stubbornness, obsessive compulsive disorder and skin picking [62–66]. These behaviours are heavily correlated with the patient's degree of obesity and hyperphagia [63]. Food seeking behavioural problems such as stealing, manipulative behaviour and self-injury are also well documented [62]. A subset of PWS patients are also diagnosed with autism spectrum disorder (ASD), attention deficit hyperactivity disorder, and psychosis which can further compound behavioural problems [67–70].

#### 2.4.4. Prader-Willi syndrome facial features

PWS patients present with distinct facial features such as narrow temple and nasal bridge, almond shaped eyes, thin upper lip and downturned mouth (collectively referred to as PWS facial features). It is reported that PWS facial features may not be present at birth and may develop over a patient's life. In addition, PWS patients have small hands and feet from GH deficiency [46] Osteoporosis leading to fractures and scoliosis is also a concern for some patients [71].

#### 2.4.5. Life expectancy

The average life expectancy for PWS patients is currently 29.5 years and the causes of mortality differ greatly between adult and child patients [4]. Cardiac, pulmonary and gastrointestinal failures are the leading causes of death. However, complications from type 2 diabetes and infections are also reported. In pediatric patients, the most common cause of mortality is respiratory failure and infections [72]. PWS patients are also vulnerable to sudden and unexpected death (SED). A myriad of studies have attempted to identify the cause of SED in PWS patients. Although no direct cause was identified, cardiovascular disease, respiratory illness and thrombosis were identified as potential risk factors for increased SED [4]. The role of CAI in SED is less certain as a few studies have found lower incidences of CAI in PWS patients [45,51] than previously reported [50].

### 2.5. Genotype–phenotype correlations

**Figure 5.**
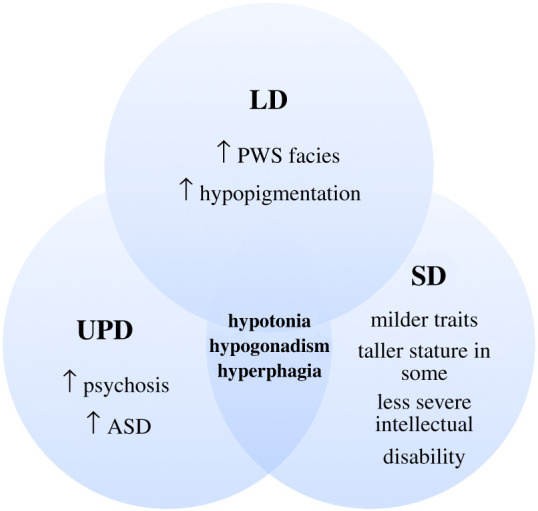
Features shared and distinct among patients with the three PWS genotypes. The hallmark traits are still shared by all three groups.

Several correlations between different PWS genetic etiologies and clinical phenotypes have been noted. More studies are required to truly elucidate the extent and significance of these correlations (figure 5).

#### 2.5.1. Effects of large deletions

Deletion patients are reported to have increased occurrence of PWS facial features [12,13], hypopigmentation (owing to deletion of one copy of Oculocutaneous Albinism II gene (*OCA2*)) [13,73,74] and intellectual disability [74]. Some studies report that type I deletion patients (BP1 to BP3) have exacerbated cases of intellectual disability and compulsory behaviour compared to type II deletion patients (BP2 to BP3) [75,76].

#### 2.5.2. Uniparental disomy

UPD patients are reported to have less PWS facial features [12,13] and little to no hypopigmentation [13,73,74]. However, they are reported to have increased risk for psychosis [67,70] and ASD [68,69]. Interestingly, PWS UPD patients are also reported to have less compulsory and behavioural problems despite these increased risks [76,77]. The differences observed between UPD and LD patients may potentially be owing to expression of two copies of *UBE3A* in UPD patients.

#### 2.5.3. Small deletions

PWS patients with SD in the proximal *SNHG14* transcript encompassing the *SNORD116* cluster are reported to demonstrate milder phenotype and absence of some clinical traits associated with PWS [17–22]. These patients still possess the hallmark traits of hypotonia, hypogonadism and hyperphagia/obesity albeit in milder forms. Some patients had normal to tall stature and absence of PWS facial features [19–22]. These findings might indicate that the absence of *SNORD116* plays a crucial role in the development of PWS phenotype. However, the absence of other surrounding regions may play a role in the severity of the phenotypes as seen in LD and UPD patients.

### 2.6. Current clinical interventions

Current treatments address a particular phenotype and are not aimed to cure the disorder.

#### 2.6.1. Hypotonia: growth hormone therapy

The natural history of PWS can be significantly improved with clinical interventions. Endocrinologists in almost all instances administer GHT for PWS patients from infancy. GHT improves the body composition by increasing muscle mass, reducing body fat while normalizing height [78,79]. It is also reported that GHT improves cognitive function and IQ scores for patients [80–82]. In adults, GHT was also shown to be beneficial by improving body composition, muscle mass and sleep-disordered breathing [83]. The impact of GHT on body mass index, hyperphagia and food-seeking behaviour is reported to be minimal but more future studies are warranted. Along with GHT, physical therapy can greatly aid PWS patients develop and maintain muscle tone in childhood and adulthood [84].

Although GHT provides tremendous benefits, each patient must be monitored carefully for potential side effects. PWS patients with CAI may experience an adrenal crisis owing to increased metabolism of cortisol [85]. Those with respiratory illness may risk hypoxemia and sleep disturbance owing to increased basal metabolism in the absence of respiratory compensation [86]. Patients with diabetes may also experience worsening of their symptoms owing to antagonism of insulin [87]. Thus endocrinologists must closely monitor each individual PWS patient based on their response to treatment. Despite these potential adverse effects, the long-term benefits of GHT are still considered to outweigh the potential risks [88,89]**.**

#### 2.6.2. Hypogonadism

Sex hormone therapy is administered and often helps patients develop secondary sex characteristics [34]. The dosage of testosterone and oestrogen has to be carefully monitored with each patient to avoid any negative side effects. With testosterone treatment, exacerbation of behavioural problems such as aggression may be noted. With oestrogen treatment, osteoporosis and possible fertility must be considered. Fertility was reported in some female PWS patients and thus sex education has to be emphasized [90,91]**.**

#### 2.6.3. Hyperphagia/obesity

Pharmaceutical interventions are currently unavailable to address hyperphagia. PWS patients are followed by both endocrinologists and dietitians. Caloric requirements are set at 60% to 80% of the age-appropriate regular daily allowance with vitamin supplementation (Miller diet) [92]. Additionally, parents and carers are coached on exercise programmes and ways to limit alternate food access by PWS patients.

#### 2.6.4. Sleep disturbances

A sleep study is recommended for most PWS patients to monitor for both potential respiratory distress and effects of GHT [86]. Interventions such as tonsillectomy and continuous positive airway pressure/bilevel positive airway pressure are available for patients with obstructive sleeping difficulties [93].

#### 2.6.5. Behavioural therapy

Speech therapy and special courses are often used to help supplement any learning difficulties [94]. Serotonin reuptake inhibitors have been reported to be effective for obsessive compulsive disorder [95] and selective serotonin reuptake inhibitors have been reported to be effective for psychosis [96].

#### 2.6.6. Experimental interventions in progress

Several experimental interventions are currently being evaluated. These interventions are often designed to address molecular deficits found in PWS. For example, PWS patients are reported to have reduced oxytocin secreting neurons which may help decrease appetite and promote satiety [97]. As a result, oxytocin therapy was developed to address this deficiency. Upon treatment, patients were reported to have reduced appetite and improved behaviour [97,98]. For hypotonia, a myostatin inhibitor may promote muscle growth and tone, and may improve metabolism [99] for hyperphagia/obesity. There are a number of other experimental interventions to treat hyperphagia/obesity as well [98,100–104].

## 3. Prader-Willi syndrome molecular genetics

The genomic imprinting of the 15q11-q13 locus is established in the germline and is controlled by the bipartite imprinting centre. PWS-IC [5–7] (figure 2) comprises a CpG island that is unmethylated on the paternal allele and methylated on the maternal allele and includes the first exon of the *SNURF*-*SNRPN* gene. The other part, the AS-IC represses the PWS-IC in the maternal germline and silences the maternal allele of chromosome 15q11-q13 in somatic tissues. The PWS-IC serves as a promoter for the approximately 600 kb long *SNRPN* transcript that serves not only as a pre-messenger RNA (mRNA) for *SNURF* and *SNRPN* but also encodes a non-coding RNA, *SNHG14*, which is a host transcript for the production of a number of both long and short non-coding RNAs such as *SPA1, SPA2, sno-lncRNAs 1–5, SNORD116, IPW, SNORD115 and UBE3A-ATS*. The proximal portion of *SNHG14*, between *SNRPN* and *IPW* is expressed in virtually all cell types in humans. The distal portion comprised of *SNORD115* and *UBE3A-ATS* is only expressed in neurons. *UBE3A-ATS*, silences paternal *UBE3A* in neurons. Thus, *UBE3A* is biallelically expressed in many tissues but is expressed exclusively from the maternal allele in neurons [105,106].

### 3.1. The *SNRPN* transcript

The bicistronic *SNRPN* transcript encodes two protein-coding genes *SNURF* and *SNRPN* as well as a approximately 600 kb long non-coding RNA (lncRNA) termed *SNHG14* [107–109]*. SNURF* is encoded by the first three exons and has unknown function while *SNRPN* is encoded by exons 4 through to 10 and produces SMN, a non-essential protein which may be involved in mRNA splicing [24,25]. The *SNHG14* non-coding RNA initiates at the upstream exons of *SNRPN* and hosts multiple RNA species with a diverse range of putative functions. For example, two clusters of small nucleolar RNAs (snoRNAs) are processed from the introns of *SNHG14*. The *SNORD116* cluster of 30 box C/D snoRNAs is closer to the PWS-IC and is expressed in most tissues while the *SNORD115* cluster of 45 box C/D snoRNAs are more distal and are expressed almost exclusively in neurons [105,106]. snoRNA ended, polyadenylated RNAs and *IPW* are processed lncRNAs with incompletely understood functions. As mentioned earlier, the distal-most portion of *SNHG14* encodes *UBE3A-ATS*, which silences paternal *UBE3A* via transcriptional interference [110]. By RNA FISH, the *SNHG14* host gene appears within nuclei as a large RNA cloud with unknown function that localizes near its site of transcription on the paternally inherited allele of chromosome 15 [109].

### 3.2. Box C/D small nucleolar RNAs

**Figure 6.**
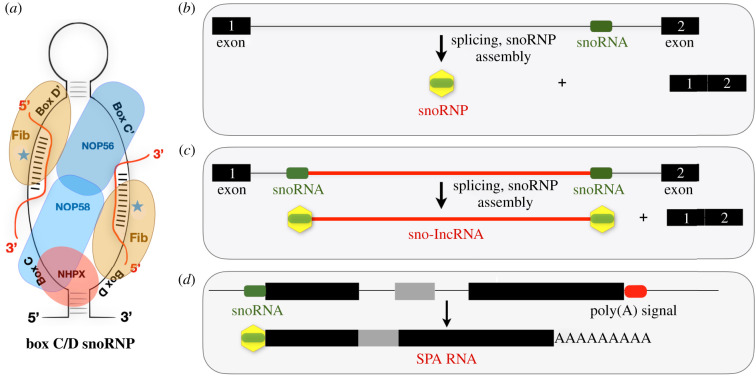
(*a*) A typical box C/D snoRNP complex including the snoRNA and associated proteins. Typical snoRNPs associate via RNA complementarity with rRNAs. Fibrillarin (Fib) catalyses the 2′-O methylation of targeted rRNA regions at the 5th bp upstream of D and D′ box as indicated by the blue star. (*b*) Box C/D snoRNAs are processed from excised introns following debranching and exonucleolytic trimming. (*c*) sno-lncRNAs are produced from introns that contain two snoRNAs. Thus, they lack 5′ cap structures and 3′ poly(A) tails but are stabilized by their terminal snoRNP components. (*d*) SPA RNAs are processed similarly to sno-lncRNAs but can be spliced and have 5′ snoRNP structures and 3′ poly(A) tails.

Of more than 100 reported post-transcriptional RNA modifications [111,112], most are found in ribosomal RNAs (rRNAs), transfer RNAs (tRNAs) and other small RNAs. However, an increasing number are becoming identified in mRNAs and lncRNAs. The most abundant RNA modifications are 2′-O methylation and pseudouridylation. These are mostly directed by snoRNAs that are usually concentrated in Cajal bodies or nucleoli where they modify either small nuclear RNAs (snRNAs) or rRNA, or participate in the processing of rRNA during ribosome biogenesis [113–115]. There are several hundred known snoRNAs, the majority of which are encoded in introns of protein-coding genes [116]. Box C/D snoRNAs are processed from excised and debranched introns by exonucleolytic trimming (figure 6*b*) [117,118] and carry out their functions in complex with specific protein components, forming ribonucleoprotein complexes (snoRNPs) consisting of the proteins NOP56, NOP58, Fibrillarin and 15.5 KD/NHPX (figure 6*a*) [113]. Box C/D snoRNAs harbour antisense sequences that basepair with target RNA substrates and guide the placement of 2′-O-methylation modification on the fifth basepair upstream of the D and D' box (figure 6*a*, blue star). 2′-O methylations directed by box C/D snoRNAs are biologically important and so far verified to exist internally only in rRNAs and snRNAs [119] and a substantial portion of known methylated sites in rRNA lie in close proximity to functional sites such as the peptidyltransferase centre, suggesting potential involvement in rRNA folding, stability and translation [119]. Interestingly, 2′-O-methylation within coding regions of artificial mRNAs has recently been reported to disrupt key steps in codon reading during cognate tRNA selection [120]. Furthermore, snoRNAs may be involved in brain development or function [121].

### 3.3. The *SNORD116* (HBII-85) cluster

The *SNORD116* cluster of box C/D snoRNAs is expressed in most tissues but their expression is much higher in the brain. The importance of this cluster to PWS pathology is very high, because all reported deletions and mutations associated with PWS lead to loss of expression from this region. A number of atypical PWS deletions have narrowed the putative PWS critical region to approximately 80 kB primarily spanning the *SNORD116* cluster (figure 4) [17–22]. While most known snoRNAs have been shown to target the modification of rRNA, the *SNORD116* snoRNAs are classified as ‘orphans' because no known targets have been identified and their sequences show no significant complementarity to rRNAs. Thus, it is crucial to identify the targets and functions of *SNORD116* snoRNAs. Mapping sites of 2′-O methylation on RNA molecules is challenging but several groups have recently developed genome-wide methods to chemically isolate and map the positions of sites of 2′-OMe [122,123]. With this technology, it will be important to identify *SNORD116* targets by comparing results from human and animal models that do or do not express *SNORD116s,* although such models may not accurately reflect the full spectrum of human phenotypes. While snoRNAs are present across a vast spectrum of organisms and often share ancient and conserved elements [113], *SNORD116* and *SNORD115*, represent blossoming groups of RNAs that may have lineage-specific molecular functions. Zhang *et al*. used a computational approach, snoSeeker, to investigate the evolution of imprinted snoRNAs across 12 placental mammalian species [124]. They discovered that the number of copies of the *SNORD116* and *SNORD115* varied widely with human and rodent lineages demonstrating the highest gains in snoRNA copies. For example, primates and rodents all possess greater than 20 copies of the *SNORD116* family, while some species such as cows and elephants possess as few as 12 and 1 copies, respectively. In addition, the birth of new snoRNA copies revealed that nucleotide substitutions occurred the most within the snoRNA sequence and not in the flanking regions.

The *SNORD116* snoRNA cluster has been further divided into groups 1–3 (*SNOG1*, *SNOG2*, *SNOG3*) based on sequence and expression heterogeneity [125]. In the hypothalamus, *SNOG1* (*SNORD116-*1 to *SNORD116-*9) is expressed most highly compared to *SNOG2* (*SNORD116-*10 to *SNORD116-*24) and *SNOG3* (*SNORD116-*25 to *SNORD116-*29). The higher reported levels of expression may actually be owing to increased stability of individual snoRNAs mediated by snoRNP complexes. In Fibrillarin RIP-Seq experiments in ovarian teratocarcinoma PA1 cells, enrichment was observed over the first third of the *SNORD116* cluster but not the latter two thirds [126]. Thus, the absence of *SNOG1* may play an important role in PWS. Kocher *et al*. investigated the sequence similarity of the *SNORD116* family across different primates and rodents [127]. Between humans and mouse, the authors noted that *SNOG1* and *SNOG2* shared greater homology, while *SNOG3* possessed smaller overlap in homology. Thus the authors proposed that the variance phenotype that is observed in murine models may be explained by the differences in *SNOG3*. As primates such as chimpanzees and macaques also share significant homology in the *SNORD116* sequence, primates may ultimately offer better models for PWS than mice.

Along with studies involving PWS SD patients, Burnett *et al*. demonstrated the importance of *SNORD116* in neurons derived from both PWS patient induced pluripotent stem cells (iPSCs) and *Snord116* knockout (KO) murine models [128]. These models showed reduced levels of *nescient helix loop helix2* (*NHLH2*) and the prohormone convertase PC1 enzyme (*PCSK1*). *Nhlh2* is reported to promote *Pcsk1* expression which in turn promotes the conversion of prohormones into mature hormones. The failure of proper hormone maturation may explain the various neuroendocrine phenotypes seen in PWS.

Several murine models of PWS have been generated to study the impact of genes relevant to the disorder. These include large deletions of the locus as well as individual deletion of *Snord116* cluster. Each model demonstrates slightly different phenotypes and severity based on genetic background of the mouse strain. However, both the LD and *Snord116* deletion models demonstrated the same phenotype to one another [129–131]. These findings further indicate that *Snord116* may be the critical gene and that its absence can be responsible for causing the majority of the PWS phenotype. Other murine models such as those with Magel2 deletions also shared some similarities with *Snord116* deletion models and thus the influence of other genes in the locus cannot entirely be ruled out [132,133].

### 3.4. Short nucleolar-long non-coding RNAs

**Figure 7.**
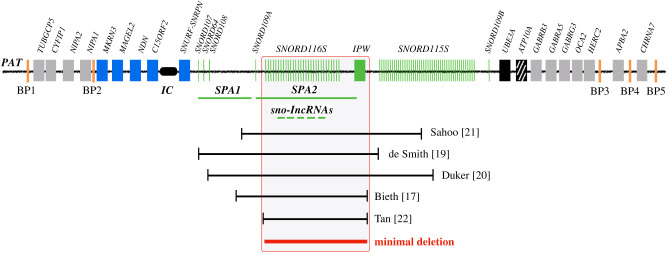
Comparison of reported microdeletions from patients with PWS features narrows the critical region to that spanning the *SNORD*116 cluster, *SPA2* and the sno-lncRNAs. Not drawn to scale.

The *SNORD116* cluster also harbours five sno-lncRNAs. These unusual RNAs have snoRNA sequences at their 5′ and 3′ caps but lack 5′-cap structures and poly(A) tails [134] (figure 6*c*). The *SNHG14* transcript hosts 5 sno-lncRNAs within the *SNORD116* cluster. *sno-lncRNA1* spans from *SNORD116-6* to *SNORD116-7*, *sno-lncRNA2* spans from *SNORD116-13* to *SNORD116-14*, *sno-lncRNA3* spans from *SNORD116-18* to *SNORD116-19*, *sno-lncRNA4* spans from *SNORD116-20* to *SNORD116-21* and *sno-lncRNA5* spans from *SNORD116-26* to *SNORD116-27*. sno-lncRNAs are strictly retained in the nucleus and accumulate at or near their site of synthesis [134]. While the complete functions of the sno-lncRNAs are not known, they harbour multiple consensus binding sites for the Fox family of splicing regulators, and have been shown to bind RBFOX2 in nuclei and together promote specific alternative splicing patterns [134]. This and the fact that all sno-lncRNAs derive from the minimal region associated with PWS (figure 7) have led to the suggestion that changes in alternative splicing may underlie at least some of the PWS clinical features. It should be noted that while sno-lncRNAs are highly expressed in human and rhesus monkeys, they are undetectable in mouse [135], perhaps partly explaining the difference in phenotypes between *SNORD116* region deletions in the different species.

### 3.5. snoRNA ended, polyadenylated RNAs

SPA RNAs are a newly described class of lncRNA that possess 5′ snoRNA cap and 3′ poly(A) tails [126]. Thus, these novel RNAs, like sno-lncRNAs, lack typical 5′-cap structures that are associated with most RNA polymerase II generated spliced transcripts. The long *SNHG14* primary transcript houses two SPAs (figure 6*d*). *SPA1* is approximately 34 000 bp in length and has seven exons. The 5′ end corresponds to *SNORD107* and the poly(A) tail forming the 3′ end is located upstream of *SNORD109A*. *SPA2* is approximately 16 000 bp in length and contains 30 exons. The 5′ cap corresponds to *SNORD109A* while the 3′ end aligns to the 3′ end of *IPW*. *SPA1* was shown to bind to the RNA binding protein TDP43, while *SPA2* binds RBFOX2 and HNRNPM [126]. Like the *SNORD116* snoRNAs and sno-lncRNAs, functions of SPA RNAs are currently not completely understood. It should be noted that while sno-lncRNAs are highly expressed in both stem cells and neurons [134], the expression of *SPA1* and *SPA2* is much greater in neurons than in stem cells [126]. *SPA1* and particularly *SPA2* are not expressed in PWS patients.

### 3.6. The *SNORD115* (HBII-52) cluster

The *SNORD115* cluster of box C/D snoRNAs consist of 45 copies and is expressed almost exclusively in neurons [107]. Unlike *SNORD116*, these snoRNAs are almost identical to one another and have an 18 bp complementarity to the serotonin 2c receptor (*HTR2C*) mRNA. This interaction has been reported to promote alternative splicing and production of mature HTR2c spliceform [107].

The *HTR2C* transcript has been reported to undergo three possible post transcriptional modifications. First, exclusion of exon 5b results in a shorter protein that is retained in the endoplasmic reticulum Second, the incorporation of exon 5b produces a fully functional receptor [136]. Third, the *HTR2C* transcript can undergo A to I editing to generate a product that includes exon 5b, but confers lower receptor activity [137,138]. It has been proposed that *SNORD115* promotes the generation of the fully functional *HTR2C* receptor transcript by blocking of splicing silencing factors and competing with deaminases for the binding of the *HTR2C* transcript [139]. Because they are expressed in some PWS patients, these snoRNAs are not likely a major cause of PWS clinical manifestations but still may worsen symptoms.

### 3.7. The partial short nucleolar RNA debate

Through RNA-Seq, several studies identified new RNA species that are produced from further processing of snoRNAs (psnoRNAs) [140,141]. The psnoRNAs are reported to have miRNA-like properties and can affect the mRNA abundance of specific transcripts. Kishore *et al*. used MBII-52 (*Snord115*) overexpression construct in a mouse neuroblastoma cell line and observed that psnoRNAs are preferentially generated over traditional canonical snoRNAs [142]. The group also report that their overexpression construct led to the expression of snoRNAs that fail to associate with classic snoRNP proteins and instead associate with splicing factors. The same group also reported that *Snord116* may undergo the same processing as *Snord115* [143].

In contrast to these above studies, other groups have failed to find the presence of abundant psnoRNAs. Bortolin-Cavaillé *et al*. found that the majority of *SNORD115* RNA species are the full length variant [144]. A smaller truncated species (larger size than psnoRNAs) was found in small abundance in mouse brain samples but completely absent in human brain samples. Both the truncated and full length *SNORD115*s were found to associate with the canonical snoRNP complex member fibrillarin. Thus these authors argued that the psnoRNAs observed in the Kishore studies were probably degradation products stemming from snoRNA overexpression. Galiveti *et al*. also failed to observe psnoRNAs across several human samples in their Northern blot experiments [145]. Consequently, the discrepancy in results from these experiments drives uncertainty about the abundance of psnoRNAs and their role in PWS.

### 3.8. SNORD107, SNORD64, SNORD108, SNORD109A, SNORD109B and IPW

Several single-copy Box C/D snoRNAs lie within the *SNHG14* transcript. These snoRNAs are *SNORD107*, *SNORD64*, *SNORD108*, *SNORD109A* and *SNORD109B*. Like the *SNORD116* snoRNAs, these appear to be orphans with no known targets in identified rRNA and other RNAs.

*IPW* is annotated as a lncRNA that was initially thought to have no functional role. In one study, however, *IPW* was reported to have a *trans*-regulatory role on the *DLK1-DIO3* imprinted region on chromosome 14 [146]. Upon overexpression of *IPW* in PWS iPSCs, the maternally expressed genes in the *DLK1-DIO3* imprinted locus was significantly downregulated. Of interest, this element also harbours a poly(A) site which serves as the termination site for the *SNHG14* transcript in stem cells [105] as well as for *SPA2* [126,147].

### 3.9. UBE3A

*UBE3A* encodes an E3 ubiquitin ligase that places a ubiquitin mark on proteins targeted for degradation by the proteasome [148,149]. It is known to target itself and RING1B, as well as several other putative proteins *in vitro*, but other targets, including bona fide *in vivo* targets, remain unknown. *UBE3A* is biallelically expressed in most tissues. However, it is imprinted in neurons by the expression of *UBE3A-ATS* from the paternal allele. It is expressed exclusively from the maternal allele in most neurons in the central nervous system [108]. The loss of function of *UBE3A* results in a separate disorder called Angelman syndrome [23].

### 3.10. ‘The left field genes' - *MKRN3*, *MAGEL2* and *NDN*

Three genomically imprinted and paternally expressed intronless genes *MKRN3*, *MAGEL2* and *NDN* lie approximately 1.3 Mb upstream of *SNPRN*. These genes may play a role in PWS based on the phenotypes that are observed upon their loss. However, some studies also report that the deletion of *MKRN3*, *MAGEL2* and *NDN* alone do not cause a PWS phenotype [150].

*MKRN3* encodes a zinc finger protein and is believed to play a role in puberty. Three frameshift mutations leading to truncation of the protein as well as a missense mutation have been reported in patients with central precocious puberty [151]. The precise function of MKRN3 is yet to be elucidated.

*MAGEL2* encodes a protein that enhances the activity of an E3 ubiquitin ligase complex [152]. It is a part of the MUST complex composed of MAGEL2, USP7 and TRIM27 [153]. The complex activates the WASH complex by ubiquitination and promotes retrograde and endosomal transport of target proteins. In addition, *MAGEL2* has also been reported to interact with proteins involved in the regulation of circadian rhythm [133].

Patients harbouring truncating mutations of *MAGEL2* have a distinct disorder called Schaaf-Yang syndrome (SYS) [154,155]. The majority of truncating mutations arises in the region of nucleotides 1990–1996 which is described as a mutational hotspot. SYS patients have some features, such as intellectual disability and hypotonia that overlap with PWS. However, they also present with ASD, contractures and other dysmorphic features which are not common to PWS. Surprisingly, deletion of *MAGEL2* has been reported to cause little to no phenotype [154,155]. These findings indicate that the truncating mutations of *MAGEL2* may encode a defective protein which acts in a dominant negative fashion [155].

The potential role of Necdin (*NDN*) has been most studied in murine models. It encodes a DNA binding protein reported to be involved in neuronal maturation by promoting cessation of cell division and promoting axonal outgrowth [156,157]. NDN has several known interacting partners. It binds the intracellular domain of the nerve growth factor receptor along with MAGEH1 and it interacts with MAGEL2 to prevent the degradation of FEZ1, an important promoter of axonal outgrowth.

*Ndn* may play an especially important role in gonadotropin releasing hormone (GnRH) neurons [158]. Overexpressed NDN protein in murine models was shown to co-immunoprecipitate with a known GnRH repressor MSX. In addition, NDN was also shown to be crucial for the generation of all subtypes of GnRH neurons and their correct projections. For this reason, *NDN* is thought to play a role in hypogonadotropic hypogonadism seen in PWS patients.

### 3.11. Other genes of unknown significance

Several genes of unknown significance lie in the 15q11-q13 locus. These genes are also imprinted and expressed from the paternal allele. *NPAP1/C15ORF2* is an imprinted and intronless gene that lies upstream of *SNRPN* and may encode a protein [159]. Two additional loci, *PWRN1* and *PWRN2* are annotated and lie upstream of *NPAP1* and the *SNRPN*. These genes appear to be non-coding [160].

## 4. Prader-Willi syndrome epigenetics

### 4.1. Epigenetic regulation of the 15q11-q13 locus

Understanding the mechanism of repression of the maternal 15q11-q13 locus is critical from a therapeutic point of view. PWS patients lack paternal contribution of the 15q11-q13 locus but possess an intact but epigenetically silent set of genes on the maternal chromosome. The regulation of gene expression at the 15q11-q13 locus is largely controlled by the PWS-IC. The PWS-IC is the master regulator and also influences DMRs at *MKRN3*, *MAGEL2* and *NDN,* which are unmethylated on the paternal allele and methylated on the maternal allele*.* The differential methylation at the PWS-IC is established in the germline. The paternal allele remains unmethylated, perhaps owing to the maintained expression from the major *SNRPN* promoter (PWS-IC) in sperm [160]. The maternal allele becomes methylated as the result of transcriptional activation of an oocyte-specific promoter(s) upstream of *SNRPN*. This transcription leads to gene body methylation as it transcribes across the PWS-IC [9,161]. The PWS-IC, and therefore the major *SNRPN* promoter is then repressed in somatic cells. How the PWS-IC influences methylation at the DMRs in *MKRN3, MAGEL2* and *NDN* is not known. This mechanism establishes the initial silencing of the maternal chromosome. In recent studies, however, the existence of separate somatic imprints in silencing the maternal allele has been discovered [162].

One of the first therapeutic proof of principle studies used the global DNA methyltransferase inhibitor 5-azadeoxycytidine (5-aza-dC) in PWS patient derived lymphoblastoid cells [163]. This compound was shown to be able to demethylate the PWS-IC leading to activation of the maternal genes. Thus the activation of the normally silent maternal genes may offer a potential therapeutic option for PWS patients.

### 4.2. The role of ZNF274 in repressing the maternal 15q11-q13 allele

Zinc Finger Protein 274 (ZNF274) is composed of a SCAN leucine rich domain, one or two Krüppel associated box (KRAB) domains, and five C2H2 zinc finger domains and also has four isoforms (a-d) using different polyadenylation signals [164]. Isoforms b and d are shorter and possess one KRAB domain. Isoforms a and c possess two KRAB domains and are longer. The DNA sequence specificity is conferred by the five zinc finger domains. ZNF274 forms a silencing complex with SET domain bifurcated 1 (SETDB1) to deposit the repressive histone mark, H3K9me3 [165]. It is reported to co-bind to multiple genomic loci with ZNF75D but also has approximately 1000 independent binding sites across the genome [166].

Cruvinel *et al*. previously discovered that ZNF274 binds to six sites within the maternal allele of *SNORD116* [167]. Along with the enrichment of ZNF274 on the maternal allele, the enrichment of H3K9me3 was also observed on the maternal allele. After knockdown of *SETDB1*, activation of maternal *SNORD116* in iPSCs from patients with PWS was seen. These results led to the proposal of a model in which ZNF274 recruits SETDB1 to maternal *SNORD116*, where it deposits H3K9me3 and contributes to repression of the maternal allele.

In subsequent studies, *ZNF274* was knocked out in iPSCs from PWS patients. Although the activation of the maternal genes was modest in iPSCs, *SNORD116* was fully activated in neural precursors and neurons differentiated from them [162]. This finding suggests that ZNF274 mediated repression of maternal *SNORD116* may represent a role for ZNF274 in maintaining a neuron-specific somatic imprint rather than a germline imprint. Interestingly, the activation of *SNORD116* initiated from the upstream, neuron-specific exons and not the PWS-IC, which remained fully methylated. These findings also reinforce the idea that this additional somatic imprint is required to maintain the repression of the maternal genes in the neural lineage.

### 4.3. G9a and GLP

G9a (Euchromatic histone lysine *N*-methyltransferase-2) and GLP (Euchromatic histone lysine *N*-methyltransferase-1) may also play an important role in establishing imprinting at the 15q11-q13 locus. G9a was previously reported to be crucial in establishing CpG methylation at the PWS-IC and catalysing the placement of H3K9me2 marks on histones in mouse embryonic stem cells [168]. Interestingly, DNA methylation was unchanged at the PWS-IC at E9.5 upon *G9a* KO. The precise interplay between H3K9me2 and DNA methylations is not known. However, it is believed that H3K9me2 marks help recruit DNA methyltransferases to the target locus and catalyse the methylation of CpG islands [169].

More recently, Kim *et al*. identified two inhibitors of G9a in a screen to identify activators of maternal *Snrpn* in mouse fibroblasts [170]. They observed partial activation of the normally silent maternal genes such as *SNORD116* in both mouse and human fibroblasts PWS models and demonstrated improved survival and growth compared to the untreated when administered by intraperitoneal injection into PWS mice. These studies revealed that the inhibition of G9a leads to decrease in H3K9me2 marks but no changes to DNA methylation at the PWS-IC. A preprint from Wu *et al*. confirmed the ability of G9a to activate maternal *SNORD116* in neural progenitors and neurons derived from human PWS iPSCs [171]. These results probably indicate that G9a plays an important role in the establishment of DNA methylation at the PWS-IC but the activation of the maternal genes are dependent on the status of H3K9 methylation, independent of the PWS-IC DNA methylation.

The relationship between G9a/GLP and ZNF274/SETDB1 and whether they share any common pathways is currently unknown. For example, SETDB1 may depend on G9a/GLP to establish H3K9me2 before it can catalyse the placement of H3K9me3. The inhibition of G9a/GLP would thus also indirectly inhibit SETDB1 by limiting the amount of available H3K9me2 substrates. It is also possible that the G9a inhibitors also directly inhibit SETDB1. Additionally, experiments to evaluate the recruitment of G9a and H3K9me2 to the human 15q11-q13 locus are necessary. It is possible that ZNF274 may co-recruit both G9a/Glp1 and SETDB1 to the locus. However, the presence of other transcription factors and recruiters for G9a/GLP cannot be ruled out.

### 4.4. SMCHD1

*Structural maintenance of chromosomes flexible hinge domain containing 1* (*SMCHD1*) encodes an epigenetic repressor. Its loss is associated with hypometyhlation and an increase in active histone modifications in its target region through manipulation of the chromatin architecture [172–175]. Both human and murine *SMCHD1* are critical in the process of X-inactivation [176,177] while murine *Smchd1* is also reported to act on other loci such as the region upstream of *Snrpn* and *Igf2r* cluster [178]. SMCHD1 is believed to be involved in the embryonic development of several structures in the head such as the eyes and the nose. However, its exact involvement in these developmental pathways is unknown. Mutations of *SMCHD1* have been implicated in disorders such as Facioscapulohumeral muscular dystrophy [179,180] and Bosma arhinia microphthalmia syndrome [181,182].

In the PWS locus, SMCHD1 was shown to be responsible for establishing the methylation imprint at *Mkrn3*, *Magel2* and *Ndn* [183]. *Smchd1* KO cells showed upregulation of these genes as well as loss of DNA methylation at the respective CpG sites. ChIP-Seq experiments also showed enrichment of H3K4me3, indicating active promoters, for *Ndn* and *Mkrn3*. SMCHD1 itself showed enrichment over the three genes as well as four other distal sites. The authors interestingly noted that four of the sites of SMCHD1 enrichment overlapped with CTCF binding sites. Thus the authors propose that SMCHD1 may antagonize CTCF mediated chromatin interactions and help establish imprinted repression of the maternal region.

The role of *SMCHD1* has not been as extensively explored in human systems. It is possible that *SMCHD1* may help establish imprinting for *MKRN3*, *MAGEL2* and *NDN* in human cells. However, the PWS-IC is clearly the master regulator controlling the imprinting of *MKRN3*, *MAGEL2* and *NDN* along with *SNRPN* because it has long been known that the deletion of the paternal PWS-IC is sufficient to cause the loss of expression of these upstream genes [164–166,184–186]. This begs the question of how DNA methylation at the PWS-IC, DNA methylation and/or histone modifications influenced by SMCHD1, and histone modifications regulated by ZNF274/SETDB1/G9A work together to repress maternal 15q11-q13.

The role of PWS-IC as the master regulator is also seen in murine models. Bressler *et al*. generated a deletion removing most of the IC in a murine model [187]. These models exhibited a similar phenotype of hypotonia and failure to thrive at birth followed by early death in 40% of the mice. The surviving mice showed developmental delay but did not develop infertility or hyperphagia/obesity. The models with a *Snrpn* segment deletion that left the IC intact did not demonstrate any phenotypes [188,189]. These studies also noted that deletion of the IC led to the loss of other paternally imprinted genes while *Snrpn* deletions sparing the IC failed to show this phenotype.

### 4.5. Implications for future therapeutics

**Figure 8.**
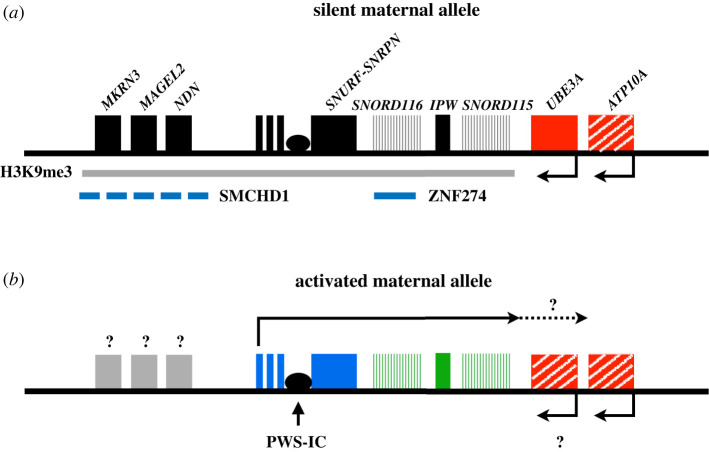
The maternal allele might serve as a therapeutic target for PWS. (*a*) The maternal allele is normally silenced owing to epigenetic silencing, some of which involves SMCHD1, ZNF274 and H3K9me3. Colour scheme is as in figure 2. (*b*) Removal of repressors such as ZNF274 and G9a have been shown to be able to activate the genes on the silent maternal chromosome, although we still do not know the full extent of activation.

The studies involving epigenetic regulators on the maternal chromosome, such as ZNF274 and G9a, may serve as therapeutic strategies for PWS (figure 8). The activation of the maternal allele may provide a permanent solution for PWS patients missing critical genes and can serve as a potential cure for the disorder. These approaches need to be further fine-tuned in the future to avoid potential genome-wide off target effects from inhibiting these epigenetic regulators. Thus future studies must explore methods to specifically exert epigenetic effects within the locus while minimizing impact at other loci. In addition, 15q11-q13 locus specific effects such as the potential silencing of *UBE3A* from over-activation of *UBE3A-ATS* also need to be monitored.

## 5. Concluding remarks

The field of PWS is constantly evolving with new discoveries driving improved outcomes for patients. Understanding the molecular underpinnings behind the clinical presentation and usage of model systems have led to the discovery of treatments that drastically improved the natural history of the disorder. For example, GHT and oxytocin therapy have greatly improved the quality of life for patients by alleviating hyperphagia and behavioural problems. However, these therapies address specific phenotypes and are not permanent cures for PWS. Recent advancements in genetic diagnosis tools have helped further pinpoint critical genes in the 15q11-q13 locus. Patients harbouring SD encompassing the *SNORD116* cluster present a majority of the PWS clinical phenotypes. Thus understanding the function of *SNORD116* including the roles of snoRNAs, sno-lncRNAs and SPA RNAs may be crucial for discovering molecular deficits that may exist between PWS and unaffected cells. In addition, the silent maternal 15q11-q13 allele may serve as an intriguing treatment option for PWS. Patients possess an intact set of genes on the maternal 15q11-q13 allele but are unable to express them owing to epigenetic silencing. Two promising silencing factors, ZNF274 and G9a, were identified in previous studies. The knockout of *ZNF274* showed robust activation of maternal *SNORD116* in neurons derived from PWS patient iPSCs. The inhibition of G9a also demonstrated activation of the maternal *SNORD116* in PWS patient fibroblasts. These promising studies provide both molecular precedence and hope that innovative therapeutics involving *SNORD116* and the maternal allele can be developed in the future.

## Data accessibility

This article has no additional data.
